# Cost-effectiveness of infection prevention and control measures for carbapenem-resistant Gram-negative bacilli: A systematic review protocol

**DOI:** 10.1136/bmjopen-2025-111472

**Published:** 2026-04-28

**Authors:** Eric Tchouaket, Fatima El-Mousawi, Stephanie Robins, Suzanne Leroux, Ibrahima Aw, Maripier Jubinville, Kelley Kilpatrick, Johanne Déry, Idrissa Beogo, Jasmin Villeneuve, Drissa Sia

**Affiliations:** 1 Canada Research Chair-Tier 2 in the Economics of Infection Prevention and Control, Université du Québec en Outaouais Département des sciences infirmières, 5 rue Saint-Joseph, Saint-Jérôme, J7Z 0B7, Québec, Canada; 2 Susan E. French Chair in Nursing Research and Innovative Practice, Ingram School of Nursing, McGill University, Montréal, Québec, Canada; 3 Département des sciences infirmières, Université du Québec en Outaouais, Saint-Jérôme, Québec, Canada; 4 School of Nursing, Faculty of Health Sciences, University of Ottawa, Ottawa, Ontario, Canada; 5 Institut national de santé publique du Québec, (INSPQ), Montréal, Québec, Canada

**Keywords:** Systematic Review, HEALTH ECONOMICS, Infection control, Health Care Costs

## Abstract

**Abstract:**

**Introduction:**

Multidrug-resistant organisms, including carbapenem-resistant Gram-negative bacilli (CRGNB), have a heavy health and economic burden in healthcare settings. Assessing the costs and clinical impact of infection prevention and control (IPC) measures targeted at CRGNB is essential to determine their cost-effectiveness and guide the allocation of limited resources. Systematic reviews of clinically effective IPC measures for carbapenem-resistant bacteria exist; however, similar reviews of economic analyses of IPC measures are lacking. Hence, the goal of this paper is to produce a protocol for a systematic review of economic evaluations of IPC measures targeting CRGNB in healthcare settings.

**Methods and analysis:**

We will query CINAHL, Medline and Embase (via Ebsco), Web of Science, Cochrane, and the grey literature for studies published between 1 January 2009 and 1 January 2026. We will include quantitative studies that describe any of the IPC strategies recommended by the WHO including hand hygiene, surveillance, contact precautions, patient isolation (single room isolation or cohorting), and environmental cleaning and that apply at least one type of economic analysis, such as cost-effectiveness, cost-benefit, cost-minimisation, cost–utility or cost–consequence. Outcomes will include net cost savings, incremental cost-effectiveness ratio, incremental cost per quality-adjusted life-year, and incremental cost per disability-adjusted life-year. Each article will be screened by three coauthors independently. One coauthor and the generative artificial intelligence chatbot Elicit will independently perform data extraction using the Consolidated Health Economic Evaluation Reporting Standards. The extractions will then be reviewed by another co-author and compiled into a single document. Two coauthors will assess the quality of the studies using three quality assessment tools: the Scottish Intercollegiate Guidelines Network checklist for economic evaluations, the Drummond critical assessment for economic evaluations, and the Cochrane Handbook for Systematic Reviews of Interventions. We will calculate and report inter-rater agreement as a percentage. We will use The Dominance Ranking Matrix by the Joanna Briggs Institute to assess whether an intervention should be favoured or rejected, or if the results are unclear. Extracted data will be synthesised, and values will be adjusted to 2026 Canadian dollars using the discount rates of 3%, 5% and 8%. Sensitivity analyses will then be performed.

**Ethics and dissemination:**

None.

**Trial registration number:**

reviewregistry1948.

**STRENGTHS AND LIMITATIONS OF THIS STUDY**

- The use of three quality assessment tools ensures the robustness of the results, coupled with the adherence to the Consolidated Health Economic Evaluation Reporting Standards checklist guidelines for data extraction, the Dominance Ranking Matrix and sensitivity analyses.
- This study will use a discounting approach, an adjustment that allows results to be compared within the same time frame.
- Restricting inclusion to English and French language publications and to available published studies may result in the omission of relevant evidence, which could limit the completeness and comparability of the findings.

## Background

 Healthcare-associated infections (HAIs) refer to infections that occur in a hospital or healthcare setting that were not present at the time of admission to the facility.^1^ On average, 1 out of 10 patients is affected by a HAI, and among these, there are 136 million patients per year affected by a multidrug-resistant organism (MDRO) globally.^1 2^ MDROs are micro-organisms, including bacteria, that show resistance to several antimicrobial agents.^3^ Carbapenem-resistant Enterobacteriaceae (CRE) are a type of MDRO that show resistance to carbapenems through different resistance mechanisms.^1^ HAIs caused by carbapenem-resistant Gram-negative bacilli (CRGNB) are associated with high morbidity, mortality and prolonged hospital stays, and have been alarmingly increasing in incidence and prevalence over the last decade.^1 4^ Once a patient is infected with CRGNB, there is a serious transmission risk in hospitals and long-term care settings, which is why strict infection prevention and control (IPC) is critical.^1^ Health agencies have established IPC strategies, such as hand hygiene, environmental cleaning and disinfection, screening and isolation, that are designed to limit the spread of infection.^1 5^ To guide policy, systematic evidence is needed on which IPC measures for CRGNB are clinically effective, how much they cost, and whether they are cost-effective compared with alternatives. Recent systematic reviews have shown the clinical effectiveness of targeted IPC strategies,^6^,^8^ but to our knowledge, no systematic reviews have demonstrated their economic benefits. The aim of this study is to conduct a systematic review of the literature to assess the economic impact and cost-effectiveness of IPC measures against CRGNB in healthcare settings and living environments, with cost values standardised through discounting.

## Methods and analysis

### Theoretical framework

The theoretical framework for the systematic review will be the WHO Guidelines for the prevention and control of carbapenem-resistant Enterobacteriaceae, Acinetobacter baumannii and *Pseudomonas aeruginosa* in healthcare facilities.^1^ This theoretical framework outlines recommendations for the prevention and control of infections with CRE, carbapenem-resistant *Acinetobacter baumannii* and carbapenem-resistant *Pseudomonas aeruginosa*. We will consider the multimodal IPC strategies including the following: (1) hand hygiene; (2) surveillance (particularly for CRE/CRGNB); (3) contact precautions; (4) patient isolation (single room isolation or cohorting) and (5) environmental cleaning.^1^ Most of these strategies were described in our previous works^9^,^12^ and are briefly summarised below.

#### Hand hygiene

Hand hygiene refers to the cleaning of the hands, wrists and forearms using water and soap, or by applying hydro-alcoholic or alcoholic antiseptic solutions.^1 13^ Proper hand hygiene is one of the most effective IPC measures, with the WHO estimating that it can reduce HAIs by 30% to 70%.^13^

#### Surveillance (particularly for CRE/CRGNB)

Surveillance for CRE/CRGNB involves systematically monitoring asymptomatic and symptomatic patients for clinical signs and symptoms of infection, combined with laboratory testing to detect and confirm carbapenem resistance in isolates obtained from clinical specimens.^1^ This process enables early detection of cases and supports timely IPC measures.

#### Contact precautions

Contact precautions are measures designed to prevent the spread of infectious agents transmitted through direct or indirect contact with a patient or their environment. They include wearing personal protective equipment such as gloves, glasses and gowns, limiting patient transport and movement, using disposable or dedicated patient-care equipment, and prioritising thorough cleaning and disinfection of rooms.^1^

#### Patient isolation (single room isolation or cohorting)

Patient isolation involves placing individuals in single rooms whenever possible to prevent the spread of infection.^1^ When single rooms are unavailable, patients carrying or infected with the same resistant pathogen may be grouped together in a shared space (cohorting).^1^

#### Environmental cleaning

Environmental cleaning is the process of cleaning and disinfecting environmental surfaces to remove visible soil and reduce the presence of microorganisms, thereby preventing the transmission of pathogens in healthcare settings.^1^

### Economic analysis

We will consider five types of economic evaluations to determine the effectiveness of IPC interventions: cost-minimisation analyses (CMA), cost-effectiveness analyses (CEA), cost–utility analyses (CUA), cost-benefit analyses (CBA) and cost–consequence analyses (CCA). These analyses were described in our previous publications.^9^,^12^

### Research objectives

The objective of this protocol is to outline a systematic review of economic evaluations of IPC measures for CRGNB using a discounting approach in order to assess the economic impact and cost-effectiveness of IPC measures.

### Eligibility criteria

We will include studies that meet the eligibility criteria defined by the Population, Intervention, Comparators and designs, Outcomes and Time framework, defined in table 1.

**Table 1:** Inclusion and exclusion criteria based on Population, Intervention, Comparators and designs, Outcomes and Time framework

	Included	Excluded
Population		
Geographic area	All countries	
Establishment	Healthcare facilities, living environments	Studies in farms or laboratories
Population	All healthcare facility patients or residents in living environments	Animals, in vitro experiments
Infections	carbapenem-resistant Enterobacteriaceae (CRE), carbapenem-resistant Gram-negative bacilli (CRGNB), any specific species/strain considered as CRE or CRGNB (example: carbapenemase-producing *Pseudomonas aeruginosa,* carbapenemase-producing *Acinetobacter baumannii*, etc)	Any other pathogen (eg, Gram positive bacteria, non-carbapenem-resistant Gram-negative bacilli, viruses, yeasts, etc)
Interventions Clinical best practices (CBPs)	Hand hygiene, surveillance for CRE/CRGNB, contact precautions, patient isolation (single room isolation or cohorting), environmental cleaning	
Comparators and designs	Quantitative studies: controlled clinical trials, randomised clinical trials, cohort studies, longitudinal studies, follow-up studies, prospective studies, retrospective studies and cross-sectional studies. Quantitative literature reviews: systematic reviews, meta-analyses, meta-syntheses. Mathematical/statistical modelling and simulations	Qualitative studies, qualitative literature reviews (scoping reviews, narrative reviews), protocols
Outcomes Types of economic evaluation Economic evaluation measures	Cost-minimisation analysis, cost-effectiveness analysis, cost–utility analysis, cost–benefit analysis or cost–consequence analysis Cost estimates of CBPs, net costs, net cost savings (savings-costs), incremental cost-effectiveness ratio (Δ costs/Δ effectiveness), incremental cost per quality-adjusted life-year, incremental cost per disability-adjusted life-year and incremental benefit–cost ratio (Δ benefits/Δ costs)	Technological assessments, purely clinical outcomes, pharmacological outcomes
Time	Studies published 1 January 2009–1 January 2026	Studies published before 2009

CBP, Clinical Best Practice.

### Population (P)

Patients and residents from all healthcare settings where IPC measures and treatments for CRGNB (including CRE, CRGNB, any specific species/strain considered as CRE or CRGNB) are applied. All countries will be included. Studies conducted with animals or in vitro will be excluded.

### Intervention (I)

We will include any intervention to prevent or control CRE/CRGNB infections in healthcare settings as outlined in WHO guidelines: hand hygiene, surveillance, contact precautions, patient isolation and environmental cleaning. CBPs and environmental interventions are included.

### Comparators and designs (C)

No comparators are defined. Quantitative studies will be included, including controlled clinical trials, randomised clinical trials, cohort studies, cross-sectional studies, longitudinal studies, follow-up studies, prospective studies, retrospective studies, systematic reviews and meta-analyses as well as mathematical/statistical modelling and simulations. Qualitative studies, and literature reviews will be excluded.

### Outcomes (O)

Outcomes will include measures of quality of life, of health and of economic outcomes through cost-effectiveness analysisanalyses (CMA, CEA, CUA, CBA, CCA) measured through the following measures: costs, net cost savings (savings-costs), incremental cost-effectiveness ratio, incremental cost per quality-adjusted life-year (QALY), incremental cost per disability-adjusted life-year (DALY) and incremental benefit–cost ratio. Any study not including at least one of these outcomes will be excluded.

### Time (T)

Research will be limited to the last seventeen years; therefore, covering studies published between 1 January 2009 and 1 January 2026. The start date was chosen because New Delhi metallo-beta-lactamase, an enzyme conferring resistance to carbapenems, was first detected in 2008, and has spread rapidly since then.^14^

### Information sources

The protocol of this systematic review has been registered in Research Registry under the following unique identifying number: reviewregistry1948. The methods were developed in accordance with the Preferred Reporting Items for Systematic Review and Meta-Analysis Protocols (PRISMA-P) 2015 statement.^15^ A PRISMA-P checklist is included as online supplemental file 1.

Searches will be conducted in CINAHL, Medline via Ebsco, Embase via Ebsco, Web of Science and Cochrane using the Boolean operators ‘AND’ and ‘OR’ (see online supplemental file 2-5, respectively). We will include articles written in English and French and published between 2009 and 2026. In addition, grey literature will be reviewed by one IPC specialist (SL) in order to capture relevant unpublished or non-indexed work.

All coauthors contributed to the determination of keywords. The CINAHL search strategy is presented in table 2.

**Table 2:** CINAHL search strategy

1	TI (Hand* OR aseptic* OR intervent* OR Programme* OR strateg* OR hygiene* OR clean* OR control OR prevention OR screen* OR wash OR protect* OR isolation OR sanitation) OR AB (Hand* OR Aseptic* OR intervent* OR Programme* OR strateg* OR hygiene* OR clean* OR control OR prevention OR screen* OR wash OR protect* OR isolation OR sanitation)
2	(MH ‘Handwashing+’) OR (MM ‘Infection Control’) OR (MM ‘Hygiene’) OR (MH ‘Patient Isolation+’)
3	TI (‘Infection control practitioner’ OR ‘infection prevention and control nurs*’ OR (IPC AND nurse) OR ‘hygiene staff’ OR ‘hygiene and sanitation’ OR ‘microbiologist’ OR (hygienist NOT dental)) OR AB (‘Infection control practitioner’ OR ‘infection prevention and control nurs*’ OR (IPC AND nurse) OR ‘hygiene staff’ OR ‘hygiene and sanitation’ OR ‘microbiologist’ OR (hygienist NOT dental))
4	TI (antibiotic OR 'antibiotic therapy’ OR carbapenem* OR 'beta-lactam’ OR 'broad-spectrum antibiotics’ OR colistin OR tigecycline OR 'carbapenem resistance’ OR 'carbapenem resistant’ OR 'carbapenem-resistant’) OR AB (antibiotic OR 'antibiotic therapy’ OR carbapenem* OR 'beta-lactam’ OR 'broad-spectrum antibiotics’ OR colistin OR tigecycline OR 'carbapenem resistance’ OR 'carbapenem resistant’ OR 'carbapenem-resistant’)
5	MM ‘Antibiotics, Combined’ OR MH ‘Antibiotics+’
6	TI (‘beta-lactam antibiotics’ OR ‘broad-spectrum antibiotics’ OR ‘antimicrobial resistance’ OR ‘antibiotic resistance’ OR ‘drug resistance’ OR ‘antibiotic therapy treatment outcomes’ OR ‘clinical outcomes’ OR ‘therapeutic efficacy’ OR ‘multidrug therapy’ OR ‘antibiotic use’ OR ‘antibiotic policy’ OR ‘antibiotic stewardship’ OR ‘empirical therapy’ OR ‘prophylactic antibiotic use’) OR AB (‘beta-lactam antibiotics’ OR ‘broad-spectrum antibiotics’ OR ‘antimicrobial resistance’ OR ‘antibiotic resistance’ OR ‘drug resistance’ OR ‘antibiotic therapy treatment outcomes’ OR ‘clinical outcomes’ OR ‘therapeutic efficacy’ OR ‘multidrug therapy’ OR ‘antibiotic use’ OR ‘antibiotic policy’ OR ‘antibiotic stewardship’ OR ‘empirical therapy’ OR ‘prophylactic antibiotic use’)
7	1 OR 2 OR 3 OR 4 OR 5 OR 6
8	TI (Cost* OR econom* OR 'econom* analysis’ OR efficienc* OR 'cost effect*’ OR 'cost util*’ OR 'cost benefit’ OR 'cost consequenc*’ OR 'cost effic*’) OR AB (Cost* OR 'econom* analysis’ OR econom* OR efficienc* OR 'cost effect*’ OR 'cost util*’ OR 'cost benefit’ OR 'cost consequenc*’ OR 'cost effic*’ or 'cost minimizat*')
9	(MH ‘Economics+’)
10	8 OR 9
11	(MM ‘Outcome Assessment’) OR (MM ‘Quality of healthcare’) OR (MM ‘Outcomes (healthcare)‘) OR (MM ‘Medical Futility’) OR (MM ‘Nursing Outcomes’) OR (MM ‘Patient-Reported Outcomes’) OR (MM ‘Treatment Outcomes’) OR (MM ‘Quality Assurance’) OR (MM ‘Quality Assessment’) OR (MM ‘Clinical Documentation Improvement’) OR (MM ‘Clinical Indicators’) OR (MM ‘Health Plan Employer Data and Information Set’) OR (MM ‘Joint Commission Core Measures’) OR (MM ‘Nursing Audit’) OR (MM ‘Outcome Assessment Information Set’) OR (MM ‘Peer Review’) OR (MM ‘Process Assessment (healthcare)‘) OR (MM ‘Utilisation Review’) OR (MM ‘Programme Evaluation’) OR (MM ‘Accountability’) OR (MM ‘Guideline Adherence’) OR (MM ‘Meaningful Use’) OR (MM ‘Professional Compliance’) OR (MM ‘Public Reporting of Healthcare Data’) OR (MM ‘Quality of Nursing Care’) OR (MM ‘Drug Efficacy’) OR (MM ‘Fatal Outcome’) OR (MM ‘Treatment Failure’) OR (MM ‘Prognosis’) OR (MM ‘Treatment Termination’) OR (MM ‘Miscellaneous Techniques’) OR (MM ‘Global Burden of Disease’) OR (MM ‘Health Services Research’) OR (MM ‘Outcomes Research’) OR (MM ‘Quality of Care Research’) OR (MM ‘Audit’)
12	(MM ‘Safety’) OR (MM ‘Patient Safety’) OR (MM ‘Accidents’) OR (MM ‘Decontamination, Hazardous Materials’) OR (MM ‘Disease Outbreaks’) OR (MM ‘Disease Transmission’) OR (MM ‘Drug Contamination’) OR (MM ‘Environmental Microbiology’) OR (MM ‘Environmental Pollution’) OR (MM ‘Exposure to Violence’) OR (MM ‘Equipment Contamination’) OR (MM ‘Equipment Reuse’) OR (MM ‘Fumigation’) OR (MM ‘Hygiene’) OR (MM ‘Infection Control’) OR (MM ‘Mandatory Reporting’) OR (MM ‘Mandatory Testing’) OR (MM ‘Sanitation’) OR (MM ‘Social Distancing’) OR (MM ‘Voluntary Reporting’) OR (MM ‘Occupational Safety’) OR (MM ‘Equipment Safety’) OR (MM ‘Patient Handling’) OR (MM ‘Seizure Precautions’) OR (MM ‘Personal Protective Equipment’) OR (MM ‘Protective Clothing’)
13	11 OR 12
14	TI ('Carbapenemase-Producing Gram Negative Bacilli’ OR 'Carbapenemase Producing Gram Negative Bacilli’ OR 'Carbapenemase-Producing Gram Negative Bacteria’ OR 'Carbapenemase Producing Gram Negative Bacteria’ OR 'carbapenemase producing enterobacteriaceae’ OR 'carbapenemase-producing enterobacteriaceae’ OR 'Carbapenem-Resistant Enterobacteriaceae’) OR AB ('Carbapenemase-Producing Gram Negative Bacilli’ OR 'Carbapenemase Producing Gram Negative Bacilli’ OR 'Carbapenemase-Producing Gram Negative Bacteria’ OR 'Carbapenemase Producing Gram Negative Bacteria’ OR 'carbapenemase producing enterobacteriaceae’ OR 'carbapenemase-producing enterobacteriaceae’ OR 'Carbapenem-Resistant Enterobacteriaceae’)
15	MM ‘Carbapenem-Resistant Enterobacteriaceae’
16	MM ‘Carbapenems+’
17	14 OR 15 OR 16
18	10 OR 13
19	7 AND 17 AND 18

### Article selection

**Figure 1:**
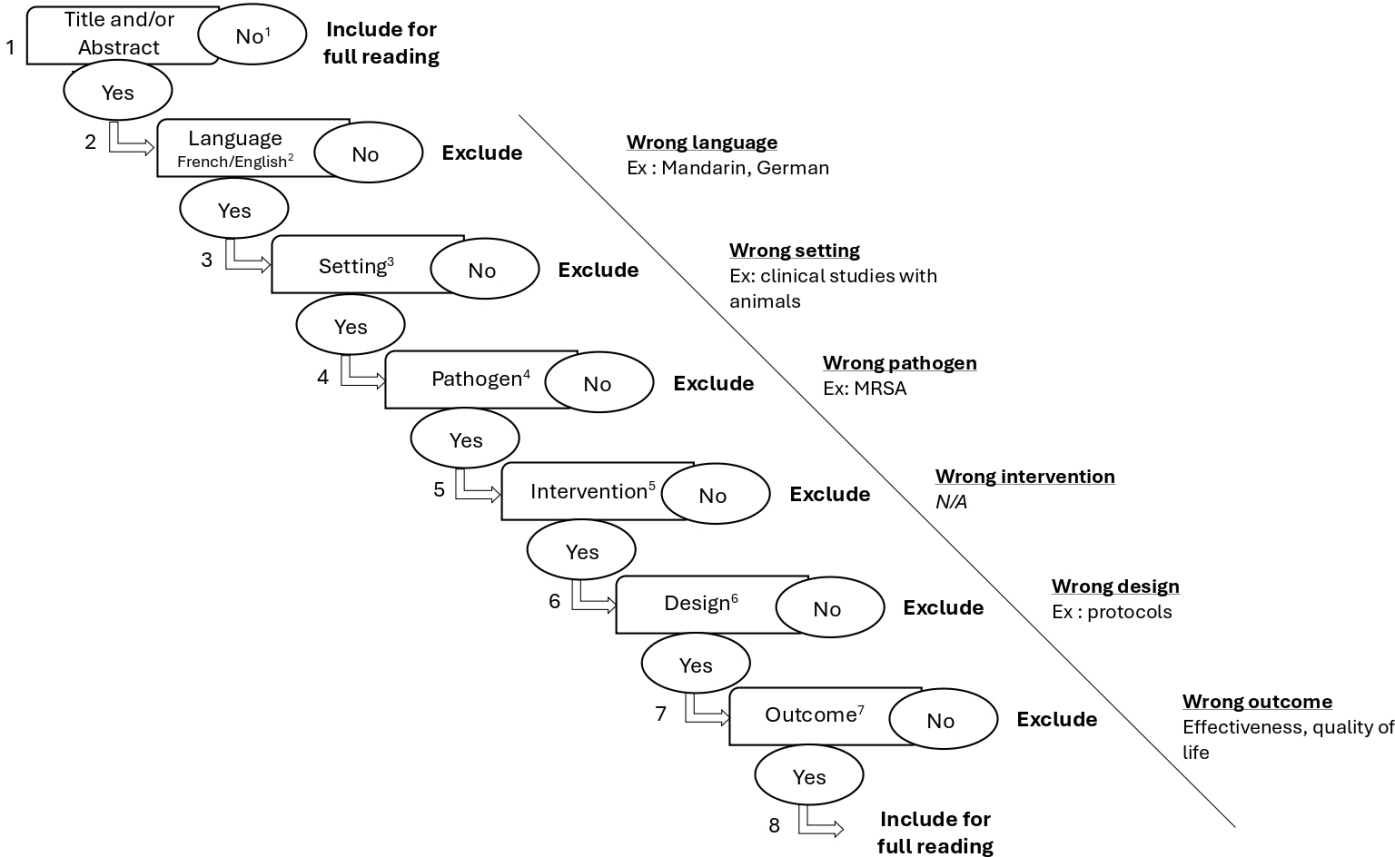
Screening algorithm. ^1^Reference: does or does not have a title and/or abstract. ^2^Language: English or French. Excluded: Spanish, Mandarin, etc. ^3^Setting: Any healthcare setting of studies conducted with humans (long-term care, acute care, etc). Excluded: Any studies conducted with animals, in vitro studies. ^4^Pathogen: Carbapenem-resistant Gram-negative bacilli. Excluded: Any other pathogen. ^5^Intervention: Clinical best practices, hand hygiene, additional precautions, screening, surveillance, environmental intervention. Excluded: antibiotic therapy, vaccination. ^6^Design: quantitative studies (controlled clinical trials, RCTs, cohort studies, longitudinal studies, follow-up studies, prospective studies, retrospective studies, cross-sectional studies) and meta-analyses, systematic reviews and studies based on mathematical and statistical modelling. Excluded: qualitative studies, qualitative literature reviews (meta-syntheses, scoping and narrative reviews), protocols, descriptive studies. ^7^Outcome: Effectiveness and efficiency measures: costs (including incremental cost-effectiveness ratio, incremental cost per quality-adjusted life-year, incremental cost per disability-adjusted life-year and the incremental cost–benefit ratio, net costs and net cost savings). MRSA, Methicillin-resistant Staphylococcus aureus; N/A, not available; RCTs, randomised clinical trials

One coauthor (FE-M) will query all databases based on the established search strategies and extract the results. All citations will be exported to the Rayyan platform^16^ where duplicates will be identified and removed, and screening will then be performed according to the inclusion and exclusion criteria. To improve reliability, prior to selection, coauthors will screen the titles and abstracts of the same 10% of articles, followed by a discussion to clarify and agree on the process. All coauthors will independently and blindly screen article titles and abstracts using an algorithm developed by our team (see figure 1) and applied elsewhere.^9^,^1217 18^ Articles will be divided equally among authors, with each article screened by two authors. In addition, one coauthor (FE-M) will screen all articles to resolve any conflicts between the paired reviewers. Articles will be included only if at least two of the three screening authors agree. The full text of retained articles will then be read entirely, and those meeting eligibility criteria will be retained for data extraction.

### Data extraction

We will create a Microsoft Excel spreadsheet to extract data from the included articles. The spreadsheet will be based on the Consolidated Health Economic Evaluation Reporting Standards (CHEERS).^19^ This spreadsheet will be used to extract data from articles such as study perspective, time horizon, type of health outcomes (eg, QALYs, DALYs), costs considered, and study findings and interpretations about the economic impact and cost-effectiveness of IPC strategies targeting CRGNB. Data extraction will be performed by one author (IA) and generative artificial intelligence chatbot (Elicit).^20^ Then, another coauthor (FE-M) will compare both extractions to solve potential conflicts and validate the extracted information and compile it in one document.

### Quality assessment

We will assess the quality of the included articles using three widely used quality assessment tools, in parallel, to ensure the robustness of the results and conclusions. The first is the checklist for economic evaluations by the Scottish Intercollegiate Guidelines Network,^21^ the second is the Drummond critical assessment for economic evaluations,^22^ and the third is the Cochrane Handbook for Systematic Reviews of Interventions.^23^ The quality assessment will be conducted independently by two coauthors (FE-M and IA) and the inter-rater agreement (IRA) will be calculated as a percentage. For each statement, a score of 1 will be assigned if raters agree and 0 if they disagree. Scores will be summed and divided by the total number of statements for each quality assessment tool to obtain the IRA percentage. To standardise evaluation and minimise interpretation biases, a third coauthor (ET) will be consulted to resolve ambiguities in statement interpretation before conducting the quality assessment. As in our previous systematic reviews,^9^,^12^ articles will then be classified according to their average score across the three tools: high (≥80%), moderate (60%–79.9%) and low (<60%).

### Data analysis and aggregation of results

The Dominance Ranking Matrix (DRM) by the Joanna Briggs Institute^24^ will be used to interpret the study and determine whether an intervention should be favoured, rejected or considered unclear. We will convert all monetary values to 2026 Canadian dollars using the Bank of Canada benchmark exchange rates and discounting rates of 3%, 5% and 8% will then be applied to bring monetary data to 2026 CAD. After conversion and discounting, sensitivity analyses will be conducted to compile and test the robustness of monetary results.

### Ethics and dissemination

This study does not require ethical approval as it involves only secondary analysis of published data and does not include individual patient data or animal subjects. This systematic review forms part of the Canada Research Chair in the Economics of Infection Prevention and Control portfolio. The results of this review will be published in a peer-reviewed journal and presented at scientific conferences.

### Implications

This protocol will lead to a review synthesising the published evidence about the economic impact of IPC measures for CRE/CRGNB. The findings will provide researchers and policymakers with current evidence-based information to support a more efficient use of limited healthcare resources and to ensure the safety and quality of care in the face of the global burden of carbapenem resistance.

### Limitations and strengths

One potential limitation of this systematic review is the exclusion of qualitative studies and literature reviews. Additionally, we restricted the search to studies published in English or French, which means relevant evidence in other languages may have been missed. As with all systematic reviews, there is also the possibility of time-lag bias, as recent or ongoing studies may not yet be published. Finally, variation in study design, outcome definitions and reporting across included studies may limit comparability and quantitative synthesis. Despite these limitations, this review will be the first, to our knowledge, to synthesise the existing evidence on the cost-effectiveness of IPC measures for CRGNB in healthcare facilities using a discounting approach. In addition, the application of three quality assessment tools, together with the adherence to the CHEERS checklist for data extraction, the DRM and sensitivity analyses, will ensure the robustness of the results.

## Supplementary material

10.1136/bmjopen-2025-111472 online supplemental file 1
