# Supplementary material for: Cost-effectiveness of infection prevention and control measures for carbapenem-resistant Gram-negative bacilli: A systematic review protocol
Source: BMJ Open. 2026 Apr 28;16(4):e111472. doi: 10.1136/bmjopen-2025-111472 (PMC13141065; doi:10.1136/bmjopen-2025-111472)
Supplement: online supplemental file 1 [file bmjopen-16-4-s001.docx]

**Supplementary File 1 - PRISMA-P 2015 Checklist**

# **This checklist has been adapted for use with systematic review protocol submissions to BioMed Central journals from Table 3 in Moher D et al**:**** Preferred reporting items for systematic review and meta-analysis protocols (PRISMA-P) 2015 statement. *Systematic Reviews* 2015 ****4****:1

# An Editorial from the Editors-in-Chief of *Systematic Reviews* details why this checklist was adapted - **Moher D, Stewart L & Shekelle P**:**** Implementing PRISMA-P: recommendations for prospective authors. *Systematic Reviews* 2016 ****5****:15

| **Section/topic** | **#** | **Checklist item** | **Information reported** | | **Line number(s)** |
| --- | --- | --- | --- | --- | --- |
|  |  |  | **Yes** | **No** |  |
| **ADMINISTRATIVE INFORMATION** | | | | | |
| **Title** | | | | | |
| Identification | 1a | Identify the report as a protocol of a systematic review |  |  | 3 |
| Update | 1b | If the protocol is for an update of a previous systematic review, identify as such |  |  |  |
| **Registration** | 2 | If registered, provide the name of the registry (e.g., PROSPERO) and registration number in the Abstract |  |  | 40 |
| **Authors** | | | | | |
| Contact | 3a | Provide name, institutional affiliation, and e-mail address of all protocol authors; provide physical mailing address of corresponding author |  |  | Anonymised |
| Contributions | 3b | Describe contributions of protocol authors and identify the guarantor of the review |  |  | 360 – 368 |
| **Amendments** | 4 | If the protocol represents an amendment of a previously completed or published protocol, identify as such and list changes; otherwise, state plan for documenting important protocol amendments |  |  |  |
| **Support** | | | | | |
| Sources | 5a | Indicate sources of financial or other support for the review |  |  | 372 – 373 |
| Sponsor | 5b | Provide name for the review funder and/or sponsor |  |  |  |
| Role of sponsor/funder | 5c | Describe roles of funder(s), sponsor(s), and/or institution(s), if any, in developing the protocol |  |  |  |
| **INTRODUCTION** | | | | | |
| **Rationale** | 6 | Describe the rationale for the review in the context of what is already known |  |  | 61 – 84 |
| **Objectives** | 7 | Provide an explicit statement of the question(s) the review will address with reference to participants, interventions, comparators, and outcomes (PICO) |  |  | 81 – 84 |
| **METHODS** | | | | | |
| **Eligibility criteria** | 8 | Specify the study characteristics (e.g., PICO, study design, setting, time frame) and report characteristics (e.g., years considered, language, publication status) to be used as criteria for eligibility for the review |  |  | 148 – 193 |
| **Information sources** | 9 | Describe all intended information sources (e.g., electronic databases, contact with study authors, trial registers, or other grey literature sources) with planned dates of coverage |  |  | 203 – 207 |
| **Search strategy** | 10 | Present draft of search strategy to be used for at least one electronic database, including planned limits, such that it could be repeated |  |  | 209 – 213 |
| ***STUDY RECORDS*** | | | | | |
| Data management | 11a | Describe the mechanism(s) that will be used to manage records and data throughout the review |  |  | 217 – 219 |
| Selection process | 11b | State the process that will be used for selecting studies (e.g., two independent reviewers) through each phase of the review (i.e., screening, eligibility, and inclusion in meta-analysis) |  |  | 219 – 227 |
| Data collection process | 11c | Describe planned method of extracting data from reports (e.g., piloting forms, done independently, in duplicate), any processes for obtaining and confirming data from investigators |  |  | 231 – 239 |
| **Data items** | 12 | List and define all variables for which data will be sought (e.g., PICO items, funding sources), any pre-planned data assumptions and simplifications |  |  | 231 – 236 |
| **Outcomes and prioritization** | 13 | List and define all outcomes for which data will be sought, including prioritization of main and additional outcomes, with rationale |  |  |  |
| **Risk of bias in individual studies** | 14 | Describe anticipated methods for assessing risk of bias of individual studies, including whether this will be done at the outcome or study level, or both; state how this information will be used in data synthesis |  |  | 243 – 255 |
| ***DATA*** | | | | | |
| **Synthesis** | 15a | Describe criteria under which study data will be quantitatively synthesized |  |  | 259 – 265 |
|  | 15b | If data are appropriate for quantitative synthesis, describe planned summary measures, methods of handling data, and methods of combining data from studies, including any planned exploration of consistency (e.g., *I* ^2^, Kendall’s tau) |  |  |  |
|  | 15c | Describe any proposed additional analyses (e.g., sensitivity or subgroup analyses, meta-regression) |  |  | 259 – 265 |
|  | 15d | If quantitative synthesis is not appropriate, describe the type of summary planned |  |  |  |
| **Meta-bias(es)** | 16 | Specify any planned assessment of meta-bias(es) (e.g., publication bias across studies, selective reporting within studies) |  |  |  |
| **Confidence in cumulative evidence** | 17 | Describe how the strength of the body of evidence will be assessed (e.g., GRADE) |  |  | 243 – 255 |

**Supplementary File 2 – Medline Search Strategy**

| 1 | AB ( (Hand* OR Aseptic* OR intervent* OR Program* OR Strateg* OR hygiene* OR Clean* OR control OR prevention OR screen* OR wash OR protect* OR isolation OR sanitation) ) OR TI ( (Hand* OR Aseptic* OR intervent* OR Program* OR Strateg* OR hygiene* OR Clean* OR control OR prevention OR screen* OR wash OR protect* OR isolation OR sanitation) ) |
| --- | --- |
| 2 | (MM "Hand Disinfection") OR (MM "Hand Hygiene+") OR (MM "Infection Control+") OR (MM "Hygiene+") OR (MM "Patient Isolation") |
| 3 | TI (“Infection control practitioner” OR “infection prevention and control nurs*” OR (IPC AND nurse) OR “hygiene staff” OR “hygiene and sanitation” OR “microbiologist” OR (hygienist NOT dental)) OR AB (“Infection control practitioner” OR “infection prevention and control nurs*” OR (IPC AND nurse) OR “hygiene staff” OR “hygiene and sanitation” OR “microbiologist” OR (hygienist NOT dental)) |
| 4 | TI (antibiotic OR 'antibiotic therapy' OR carbapenem* OR 'beta-lactam' OR 'broad-spectrum antibiotics' OR colistin OR tigecycline OR 'carbapenem resistance' OR 'carbapenem resistant' OR 'carbapenem-resistant') OR AB (antibiotic OR 'antibiotic therapy' OR carbapenem* OR 'beta-lactam' OR 'broad-spectrum antibiotics' OR colistin OR tigecycline OR 'carbapenem resistance' OR 'carbapenem resistant' OR 'carbapenem-resistant') |
| 5 | (MM "Anti-Bacterial Agents+") OR (MM "beta Lactam Antibiotics+") |
| 6 | TI ("beta-lactam antibiotics" OR "broad-spectrum antibiotics" OR "antimicrobial resistance" OR "antibiotic resistance" OR "drug resistance" OR "antibiotic therapy treatment outcomes" OR "clinical outcomes" OR "therapeutic efficacy" OR "multidrug therapy" OR "antibiotic use" OR "antibiotic policy" OR "antibiotic stewardship" OR "empirical therapy" OR "prophylactic antibiotic use") OR AB ("beta-lactam antibiotics" OR "broad-spectrum antibiotics" OR "antimicrobial resistance" OR "antibiotic resistance" OR "drug resistance" OR "antibiotic therapy treatment outcomes" OR "clinical outcomes" OR "therapeutic efficacy" OR "multidrug therapy" OR "antibiotic use" OR "antibiotic policy" OR "antibiotic stewardship" OR "empirical therapy" OR "prophylactic antibiotic use") |
| 7 | 1 OR 2 OR 3 OR 4 OR 5 OR 6 |
| 8 | TI ( Cost* OR econom* OR 'econom* analysis' OR efficienc* OR 'cost effect*' OR 'cost util*' OR 'cost benefit' OR 'cost consequenc*' OR 'cost effic*' ) OR AB ( Cost* OR 'econom* analysis' OR econom* OR efficienc* OR 'cost effect*' OR 'cost util*' OR 'cost benefit' OR 'cost consequenc*' OR 'cost effic*' or 'cost minimizat***'**) |
| 9 | (MM "Economics+") |
| 10 | 8 OR 9 |
| 11 | (MM "Outcome Assessment, Health Care+") OR (MM "Outcome and Process Assessment, Health Care+") OR (MM "Quality of Health Care+") OR (MM "Medical Futility") OR (MM "Patient Outcome Assessment+") OR (MM "Patient Reported Outcome Measures+") OR (MM "Treatment Outcome+") OR (MM "Quality Assurance, Health Care+") OR (MM "Quality Improvement+") OR (MM "Quality Indicators, Health Care+") OR (MM "Health Plan Implementation") OR (MM "Nursing Audit") OR (MM "Guideline Adherence") OR (MM "Meaningful Use") OR (MM "Public Reporting of Healthcare Data") OR (MM "Fatal Outcome") OR (MM "Treatment Failure+") OR (MM "Prognosis+") OR (MM "Global Burden of Disease") OR (MM "Health Services Research+") |
| 12 | (MM "Personal Protective Equipment+") OR (MM "Protective Clothing+") OR (MH "Safety") OR (MH "Patient Safety") OR (MH "Accidents") OR (MH "Decontamination") OR (MH "Disease Outbreaks") OR (MH "Disease Transmission, Infectious") OR (MH "Drug Contamination") OR (MH "Environmental Microbiology") OR (MH "Environmental Pollution") OR (MH "Equipment Reuse") OR (MH "Equipment Contamination") OR (MH "Fumigation") OR (MH "Food Safety") OR (MH "Hygiene") OR (MH "Infection Control") OR (MH "Mandatory Reporting") OR (MH "Mandatory Testing") OR (MH "Sanitation") OR (MH "Physical Distancing") OR (MH "Occupational Health") OR (MH "Equipment Safety") |
| 13 | 11 OR 12 |
| 14 | TI ('Carbapenemase-Producing Gram Negative Bacilli' OR 'Carbapenemase Producing Gram Negative Bacilli' OR 'Carbapenemase-Producing Gram Negative Bacteria' OR 'Carbapenemase Producing Gram Negative Bacteria' OR 'carbapenemase producing enterobacteriaceae' OR 'carbapenemase-producing enterobacteriaceae' OR 'Carbapenem-Resistant Enterobacteriaceae') OR AB ('Carbapenemase-Producing Gram Negative Bacilli' OR 'Carbapenemase Producing Gram Negative Bacilli' OR 'Carbapenemase-Producing Gram Negative Bacteria' OR 'Carbapenemase Producing Gram Negative Bacteria' OR 'carbapenemase producing enterobacteriaceae' OR 'carbapenemase-producing enterobacteriaceae' OR 'Carbapenem-Resistant Enterobacteriaceae') |
| 15 | (MM "Carbapenems+") OR (MM "Carbapenem-Resistant Enterobacteriaceae") |
| 16 | 14 OR 15 |
| 17 | 10 OR 13 |
| 18 | 7 AND 16 AND 17 |

**Supplementary File 3 – Embase Search Strategy**

| 1 | hand*:ab,ti,kw OR aseptic*:ab,ti,kw OR intervent*:ab,ti,kw OR program*:ab,ti,kw OR strateg*:ab,ti,kw OR hygiene*:ab,ti,kw OR clean*:ab,ti,kw OR control:ab,ti,kw OR prevention:ab,ti,kw OR screen*:ab,ti,kw OR wash:ab,ti,kw OR protect*:ab,ti,kw OR isolation:ab,ti,kw OR sanitation:ab,ti,kw |
| --- | --- |
| 2 | hand disinfection'/exp OR 'hand washing'/exp OR 'infection control'/exp OR 'hygiene'/exp OR 'patient isolation'/exp |
| 3 | infection control practitioner':ab,ti,kw OR 'infection prevention and control nurs*':ab,ti,kw OR (ipc:ab,ti,kw AND nurse:ab,ti,kw) OR 'hygiene staff':ab,ti,kw OR 'hygiene and sanitation':ab,ti,kw OR 'microbiologist':ab,ti,kw OR (hygienist:ab,ti,kw NOT dental:ab,ti,kw) |
| 4 | antibiotic:ab,ti,kw OR 'antibiotic therapy':ab,ti,kw OR carbapenem*:ab,ti,kw OR 'beta-lactam':ab,ti,kw OR 'broad-spectrum antibiotics':ab,ti,kw OR colistin:ab,ti,kw OR tigecycline:ab,ti,kw OR 'carbapenem resistance':ab,ti,kw OR 'carbapenem resistant':ab,ti,kw OR 'carbapenem-resistant':ab,ti,kw |
| 5 | antibiotic therapy'/exp OR 'beta lactam antibiotic'/exp |
| 6 | beta-lactam antibiotics':ab,ti,kw OR 'broad-spectrum antibiotics':ab,ti,kw OR 'antimicrobial resistance':ab,ti,kw OR 'antibiotic resistance':ab,ti,kw OR 'drug resistance':ab,ti,kw OR 'antibiotic therapy treatment outcomes':ab,ti,kw OR 'clinical outcomes':ab,ti,kw OR 'therapeutic efficacy':ab,ti,kw OR 'multidrug therapy':ab,ti,kw OR 'antibiotic use':ab,ti,kw OR 'antibiotic policy':ab,ti,kw OR 'antibiotic stewardship':ab,ti,kw OR 'empirical therapy':ab,ti,kw OR 'prophylactic antibiotic use':ab,ti,kw |
| 7 | #1 OR #2 OR #3 OR #4 OR #5 OR #6 |
| 8 | cost*:ab,ti,kw OR econom*:ab,ti,kw OR 'econom* analysis':ab,ti,kw OR efficienc*:ab,ti,kw OR 'cost effect*':ab,ti,kw OR 'cost util*':ab,ti,kw OR 'cost benefit':ab,ti,kw OR 'cost consequenc*':ab,ti,kw OR 'cost effic*':ab,ti,kw |
| 9 | economic aspect'/exp |
| 10 | #8 OR #9 |
| 11 | outcome assessment':ab,ti,kw OR 'quality of health care':ab,ti,kw OR 'medical futility':ab,ti,kw OR 'nursing outcomes':ab,ti,kw OR 'patient-reported outcomes':ab,ti,kw OR 'treatment outcomes':ab,ti,kw OR 'quality assurance':ab,ti,kw OR 'quality assessment':ab,ti,kw OR 'clinical documentation improvement':ab,ti,kw OR 'clinical indicators':ab,ti,kw OR 'health plan employer data and information set':ab,ti,kw OR 'joint commission core measures':ab,ti,kw OR 'nursing audit':ab,ti,kw OR 'outcome assessment information set':ab,ti,kw OR 'peer review':ab,ti,kw OR 'process assessment':ab,ti,kw OR 'utilization review':ab,ti,kw OR 'program evaluation':ab,ti,kw OR 'accountability':ab,ti,kw OR 'guideline adherence':ab,ti,kw OR 'meaningful use':ab,ti,kw OR 'professional compliance':ab,ti,kw OR 'public reporting of healthcare data':ab,ti,kw OR 'quality of nursing care':ab,ti,kw OR 'drug efficacy':ab,ti,kw OR 'fatal outcome':ab,ti,kw OR 'treatment failure':ab,ti,kw OR 'prognosis':ab,ti,kw OR 'treatment termination':ab,ti,kw OR 'miscellaneous techniques':ab,ti,kw OR 'global burden of disease':ab,ti,kw OR 'health services research':ab,ti,kw OR 'outcomes research':ab,ti,kw OR 'quality of care research':ab,ti,kw OR 'audit':ab,ti,kw |
| 12 | safety':ab,ti,kw OR 'patient safety':ab,ti,kw OR 'accidents':ab,ti,kw OR 'decontamination':ab,ti,kw OR 'hazardous materials':ab,ti,kw OR 'disease outbreaks':ab,ti,kw OR 'disease transmission':ab,ti,kw OR 'drug contamination':ab,ti,kw OR 'environmental microbiology':ab,ti,kw OR 'environmental pollution':ab,ti,kw OR 'equipment contamination':ab,ti,kw OR 'equipment reuse':ab,ti,kw OR 'hygiene':ab,ti,kw OR 'infection control':ab,ti,kw OR 'sanitation':ab,ti,kw OR 'social distancing':ab,ti,kw OR 'equipment safety':ab,ti,kw OR 'environmental safety':ab,ti,kw OR 'individual safety':ab,ti,kw OR 'personal protective equipment':ab,ti,kw OR 'protective clothing':ab,ti,kw OR 'respiratory protective devices':ab,ti,kw OR 'masks':ab,ti,kw |
| 13 | #11 OR #12 |
| 14 | carbapenemase-producing gram negative bacilli':ab,kw,ti OR 'carbapenemase producing gram negative bacilli':ab,kw,ti OR 'carbapenemase-producing gram negative bacteria':ab,kw,ti OR 'carbapenemase producing gram negative bacteria':ab,kw,ti OR 'carbapenemase producing enterobacteriaceae':ab,kw,ti OR 'carbapenemase-producing enterobacteriaceae':ab,kw,ti OR 'carbapenem-resistant enterobacteriaceae':ab,kw,ti |
| 15 | carbapenem-resistant enterobacteriaceae'/exp OR 'carbapenem'/exp |
| 16 | #14 OR #15 |
| 17 | #10 OR #13 |
| 18 | #7 AND #16 AND #17 |

**Supplementary File 4 – Web of Science Search Strategy**

| 1 | TS=(Hand* OR Aseptic* OR intervent* OR Program* OR Strateg* OR hygiene* OR Clean* OR control OR prevention OR screen* OR wash OR protect* OR isolation OR sanitation) |
| --- | --- |
| 2 | TS=("Infection control practitioner" OR "infection prevention and control nurs*" OR "IPC AND nurse" OR "hygiene staff" OR "hygiene and sanitation" OR "microbiologist" OR "hygienist NOT dental") |
| 3 | TS=("beta-lactam antibiotics" OR "broad-spectrum antibiotics" OR "antimicrobial resistance" OR "antibiotic resistance" OR "drug resistance" OR "antibiotic therapy treatment outcomes" OR "clinical outcomes" OR "therapeutic efficacy" OR "multidrug therapy" OR "antibiotic use" OR "antibiotic policy" OR "antibiotic stewardship" OR "empirical therapy" OR "prophylactic antibiotic use") |
| 4 | 1 OR 2 OR 3 |
| 5 | TS=(Cost* OR econom* OR "economic analysis" OR efficienc* OR "cost effect*" OR "cost util*" OR "cost benefit" OR "cost consequenc*" OR "cost effic*") |
| 6 | TS=(("Outcome Assessment" OR "Quality of Health Care" OR "Medical Futility" OR "Nursing Outcomes" OR "Patient-Reported Outcomes" OR "Treatment Outcomes" OR "Quality Assurance" OR "Quality Assessment" OR "Clinical Documentation Improvement" OR "Clinical Indicators" OR "Health Plan Employer Data and Information Set" OR "Joint Commission Core Measures" OR "Nursing Audit" OR "Outcome Assessment Information Set" OR "Peer Review" OR "Process Assessment" OR "Utilization Review" OR "Program Evaluation" OR "Accountability" OR "Guideline Adherence" OR "Meaningful Use" OR "Professional Compliance" OR "Public Reporting of Healthcare Data" OR "Quality of Nursing Care" OR "Drug Efficacy" OR "Fatal Outcome" OR "Treatment Failure" OR "Prognosis" OR "Treatment Termination" OR "Miscellaneous Techniques" OR "Global Burden of Disease" OR "Health Services Research" OR "Outcomes Research" OR "Quality of Care Research" OR "Audit") ) |
| 7 | TS=(("Safety" OR "Patient Safety" OR "Accidents" OR "Decontamination" OR "Hazardous Materials" OR "Disease Outbreaks" OR "Disease Transmission" OR "Drug Contamination" OR "Environmental Microbiology" OR "Environmental Pollution" OR "Equipment Contamination" OR "Equipment Reuse" OR "Hygiene" OR "Infection Control" OR "Sanitation" OR "Social Distancing" OR "Equipment Safety" OR "Environmental Safety" OR "Individual Safety" OR "Personal Protective Equipment" OR "Protective Clothing" OR "Respiratory Protective Devices" OR "Masks") ) |
| 8 | 6 OR 7 |
| 9 | TI=("Carbapenemase-Producing Gram Negative Bacilli" OR "Carbapenemase Producing Gram Negative Bacilli" OR "Carbapenemase-Producing Gram Negative Bacteria" OR "Carbapenemase Producing Gram Negative Bacteria" OR "carbapenemase producing enterobacteriaceae" OR "carbapenemase-producing enterobacteriaceae" OR "Carbapenem-Resistant Enterobacteriaceae") OR AB=("Carbapenemase-Producing Gram Negative Bacilli" OR "Carbapenemase Producing Gram Negative Bacilli" OR "Carbapenemase-Producing Gram Negative Bacteria" OR "Carbapenemase Producing Gram Negative Bacteria" OR "carbapenemase producing enterobacteriaceae" OR "carbapenemase-producing enterobacteriaceae" OR "Carbapenem-Resistant Enterobacteriaceae") |
| 10 | 5 OR 8 |
| 11 | 4 AND 9 AND 10 |

**Supplementary File 5 – Cochrane Search Strategy**

| 1 | (Hand* OR Aseptic* OR intervent* OR ("program" NEXT strateg*) OR hygiene* OR Clean* OR control OR prevention OR ("screen" NEXT protect*) OR wash OR protect* OR isolation OR sanitation) in Title, Abstract, Keywords |
| --- | --- |
| 2 | ("Infection control practitioner" OR ("infection prevention and control" NEXT nurs*) OR (IPC AND nurse) OR "hygiene staff" OR "hygiene and sanitation" OR "microbiologist" OR (hygienist NOT dental)) in Title, Abstract, Keywords |
| 3 | MeSH descriptor: [Hand Disinfection] explode all trees |
| 4 | MeSH descriptor: [Infection Control] explode all trees |
| 5 | MeSH descriptor: [Hygiene] explode all trees |
| 6 | MeSH descriptor: [Patient Isolation] explode all trees |
| 7 | MeSH descriptor: [Sanitation] explode all trees |
| 8 | (antibiotic OR "antibiotic therapy" OR carbapenem* OR "beta-lactam" OR "broad-spectrum antibiotics" OR colistin OR tigecycline OR "carbapenem resistance" OR "carbapenem resistant" OR "carbapenem-resistant") in Title, Abstract, Keywords |
| 9 | ("beta-lactam antibiotics" OR "broad-spectrum antibiotics" OR "antimicrobial resistance" OR "antibiotic resistance" OR "drug resistance" OR "antibiotic therapy treatment outcomes" OR "clinical outcomes" OR "therapeutic efficacy" OR "multidrug therapy" OR "antibiotic use" OR "antibiotic policy" OR "antibiotic stewardship" OR "empirical therapy" OR "prophylactic antibiotic use") in Title, Abstract, Keywords |
| 10 | #1 OR #2 #3 #4 OR #5 OR #6 OR #8 OR #9 |
| 11 | (Cost* OR econom* OR ("economic" NEXT analysis) OR efficienc* OR ("cost" NEXT effect*) OR ("cost" NEXT util*) OR ("cost" NEXT benefit) OR ("cost" NEXT consequenc*) OR ("cost" NEXT effic*) OR ("cost" NEXT minimizat*)) in Title, Abstract, Keywords |
| 12 | MeSH descriptor: [Economics] explode all trees |
| 13 | ( price OR prices OR pricing OR expenditure OR expenditures OR expense OR expenses OR finan* OR fees OR charges OR budget OR budgets OR cost* OR  ("economic" NEXT analysis) OR efficienc*):ti,ab,kw |
| 14 | MeSH descriptor: [Costs and Cost Analysis] explode all trees |
| 15 | MeSH descriptor: [Cost-Benefit Analysis] explode all trees |
| 16 | #11 OR #12 OR #13 OR #14 OR #15 |
| 17 | MeSH descriptor: [Outcome Assessment, Health Care] explode all trees |
| 18 | MeSH descriptor: [Quality of Health Care] explode all trees |
| 19 | MeSH descriptor: [Medical Futility] explode all trees |
| 20 | MeSH descriptor: [Patient Reported Outcome Measures] explode all trees |
| 21 | MeSH descriptor: [Treatment Outcome] explode all trees |
| 22 | MeSH descriptor: [Quality Assurance, Health Care] explode all trees |
| 23 | MeSH descriptor: [Nursing Audit] explode all trees |
| 24 | MeSH descriptor: [Patient Outcome Assessment] explode all trees |
| 25 | MeSH descriptor: [Peer Review] explode all trees |
| 26 | MeSH descriptor: [Process Assessment, Health Care] explode all trees |
| 27 | MeSH descriptor: [Utilization Review] explode all trees |
| 28 | MeSH descriptor: [Program Evaluation] explode all trees |
| 29 | MeSH descriptor: [Guideline Adherence] explode all trees |
| 30 | MeSH descriptor: [Meaningful Use] explode all trees |
| 31 | MeSH descriptor: [Public Reporting of Healthcare Data] explode all trees |
| 32 | MeSH descriptor: [Fatal Outcome] explode all trees |
| 33 | MeSH descriptor: [Treatment Failure] explode all trees |
| 34 | MeSH descriptor: [Prognosis] explode all trees |
| 35 | MeSH descriptor: [Global Burden of Disease] explode all trees |
| 36 | MeSH descriptor: [Health Services Research] explode all trees |
| 37 | MeSH descriptor: [Safety] explode all trees |
| 38 | MeSH descriptor: [Patient Safety] explode all trees |
| 39 | MeSH descriptor: [Decontamination] explode all trees |
| 40 | MeSH descriptor: [Disease Outbreaks] explode all trees |
| 41 | MeSH descriptor: [Disease Transmission, Infectious] explode all trees |
| 42 | MeSH descriptor: [Environmental Microbiology] explode all trees |
| 43 | MeSH descriptor: [Environmental Pollution] in all MeSH products |
| 44 | MeSH descriptor: [Equipment Contamination] explode all trees |
| 45 | MeSH descriptor: [Equipment Reuse] explode all trees |
| 46 | MeSH descriptor: [Hygiene] explode all trees |
| 47 | MeSH descriptor: [Infection Control] explode all trees |
| 48 | MeSH descriptor: [Sanitation] explode all trees |
| 49 | MeSH descriptor: [Physical Distancing] explode all trees |
| 50 | MeSH descriptor: [Occupational Health] explode all trees |
| 51 | MeSH descriptor: [Equipment Safety] explode all trees |
| 52 | MeSH descriptor: [Emergency Medical Services] explode all trees |
| 53 | MeSH descriptor: [Personal Protective Equipment] explode all trees |
| 54 | MeSH descriptor: [Protective Clothing] explode all trees |
| 55 | MeSH descriptor: [Respiratory Protective Devices] explode all trees |
| 56 | MeSH descriptor: [Masks] explode all trees |
| 57 | #17 OR #18 OR #19 OR #20 OR #21 OR #22 OR #23 OR #24 OR #25 OR #26 OR #27 OR #28 OR #29 OR #30 OR #31 OR #32 OR #33 OR #34 OR #35 OR #36 OR #37 OR #38 #39 OR #40 OR #41 OR #42 OR #43 #44 OR #45 #46 OR #47 #48 OR #49 OR #50 OR #51 OR #52 OR #53 OR #54 OR #55 OR #56 |
| 58 | (  "Carbapenemase-Producing Gram Negative Bacilli" OR   "Carbapenemase Producing Gram Negative Bacilli" OR   "Carbapenemase-Producing Gram Negative Bacteria" OR   "Carbapenemase Producing Gram Negative Bacteria" OR   "carbapenemase producing enterobacteriaceae" OR   "carbapenemase-producing enterobacteriaceae" OR   "Carbapenem-Resistant Enterobacteriaceae" ):ti,ab,kw |
| 59 | MeSH descriptor: [Carbapenems] explode all trees |
| 60 | MeSH descriptor: [Carbapenem-Resistant Enterobacteriaceae] explode all trees |
| 61 | #58 OR #59 OR #60 |
| 62 | #16 OR #57 |
| 63 | #10 AND #61 AND #62 |
