# Supplementary material for: Cost-effectiveness of infection prevention and control measures for carbapenem-resistant Gram-negative bacilli: A systematic review protocol
Source: BMJ Open. 2026 Apr 28;16(4):e111472. doi: 10.1136/bmjopen-2025-111472 (PMC13141065; doi:10.1136/bmjopen-2025-111472)
Supplement: Supplementary file 2 [file bmjopen-2025-111472.reviewer_comments.pdf]

PEER REVIEW HISTORY

BMJ Open publishes all reviews undertaken for accepted manuscripts. Reviewers are asked to complete a checklist review form and are provided with free text boxes to elaborate on their assessment. These free text comments are reproduced below.

ARTICLE DETAILS

Title (Provisional)

Cost-effectiveness of Infection Prevention and Control Measures for Carbapenemase-Resistant Gram-Negative Bacilli: A Systematic Review Protocol

Authors

Tchouaket, Eric Nguemeleu; El-Mousawi, Fatima; Robins, Stephanie; Leroux, Suzanne; Aw, Ibrahima; Jubinville, Maripier; Kilpatrick, Kelley; Déry, Johanne; Beogo, Idrissa; Villeneuve, Jasmin; Sia, Drissa

---

VERSION 1 - REVIEW

**Reviewer**	**1**
**Name**	**YU, Zhenwei**
**Affiliation**	**Zhejiang University School of Medicine Sir Run Run Shaw Hospital, Department of Pharmacy**
**Date**	**23-Oct-2025**
**COI**	**None**

Thanks for giving the opportunity to review this manuscript. This manuscript has presented a protocol of systematic review, which focuses on the economic outcome of infection prevention and control measure for carbapenemase-producing gram-negative bacilli (CPGNB). Infection control is truly an important issue and its economic burden and outcome are concerned. However, this systematic review has some major concerns.

1 This systematic review only focuses on CPGNB, however, other pathogens, such as ESBL-E, also need prevention. Moreover, the prevention measure for CPGNB is not unique, it is almost same as measures for other pathogens, and it would also be effective for other pathogens. A cited protocol, [11]Protocol for a systematic review of economic analyses of nosocomial infection prevention and control interventions in OECD hospitals. doi: 10.1136/bmjopen-2020-037765 is much more reasonable. And the results would be indifferent.

2 The rational of selecting CPGNB rather than CRGNB. Carbapenemase is an important mechanism of carbapenem resistance for GNB, however, not all carbapenemase-producing

GNB are carbapenem resistant. It may be difficult to distinguish the mechanism of resistance in clinical practice, especially for source-limiting areas.

3 Inclusion criteria of population in table 1 and in maintext are conflict. Table 1 seems to specify the infection type.

4 Sensitivity analyses in the main text is as simple as in abs. No subgroup analysis was planned.

**Reviewer**	**2**
**Name**	**Manoukian, Sarkis**
**Affiliation**	**Glasgow Caledonian University, School of Health and Life Sciences**
**Date**	**08-Dec-2025**
**COI**	**None**

I have reviewed this protocol and even though the work looks promising and useful I have some serious concerns about the planned review

1) The decision to restrict the review to carbapenemase-producing organisms (CPE), excluding broader carbapenem-resistant (CRE) studies, requires justification. There is a potential risk that the exclusion could lead to the omission of relevant economic evaluations where phenotypic resistance is the primary cost driver, regardless of the underlying genotype. It would be prudent to expand the search and include CRE especially given a gen AI tool will be used to speed things up and there are not many cost-effectiveness studies in this literature unless there is a compelling reason not to do this.

2) You plan to to apply discount rates to aggregate monetary values whilst assuming health effects remain stable is inconsistent with standard health economic guidelines for time horizons exceeding one year. This plan needs revision to ensure that both costs and outcomes are discounted appropriately and consistently. Also the current plan to apply a discount rate to a reported aggregate cost is confusing, what are you trying to achieve given discounting is a function of time within the model's horizon and applying a flat discount rate to a static cost figure sounds mathematically meaningless?

3) The proposed approach of averaging scores across three different assessment tools should be discouraged, as the proposed summary scores can mask critical methodological flaws in specific domains. Furthermore, applying the Cochrane Risk of Bias tool—designed for clinical trials—to decision-analytic models risks penalising high-quality modelling studies. I'd like to see how these concerns can be addressed

4) The plan to synthesise findings from multiple countries using a single Dominance Ranking Matrix requires clarification regarding how differences in unit costs, currency purchasing

power, and health system thresholds will be handled. Without adjusting for these structural variations, direct ranking of ICERs across different jurisdictions are very likely to yield misleading conclusions which would diminish the utility of your review greatly.

VERSION 1 - AUTHOR RESPONSE

Reviewer 1

Reviewer's comments	Responses
This systematic review only focuses on CPGNB, however, other pathogens, such as ESBL-E, also need prevention. Moreover, the prevention measure for CPGNB is not unique, it is almost same as measures for other pathogens, and it would also be effective for other pathogens. A cited protocol, [11]Protocol for a systematic review of economic analyses of nosocomial infection prevention and control interventions in OECD hospitals. doi: 10.1136/bmjopen-2020-037765 is much more reasonable. And the results would be indifferent.	Thank you for this comment, we agree with your point. We will make our review more comprehensive and include CRGNB, regardless of their mechanism of resistance. Please refer to our "Background"; and "Methods and Analysis" sections.
The rational of selecting CPGNB rather than CRGNB. Carbapenemase is an important mechanism of carbapenem resistance for GNB, however, not all carbapenemase-producing GNB are carbapenem resistant. It may be difficult to distinguish the mechanism of resistance in clinical practice, especially for source-limiting areas.	We agree as mentioned earlier, we will include CRGNB.
Inclusion criteria of population in table 1 and in maintext are conflict. Table 1 seems to specify the infection type.	We have added infection type to the text, please refer to the "Population" section.
Sensitivity analyses in the main text is as simple as in abs. No subgroup analysis was planned.	We are aware of this, once we have the data, we will assess if subgroup analysis is warranted/possible/necessary.

Commented [EMF1]: I'm not sure to understand this comment

Reviewer 2

Reviewer's comments	Responses
The decision to restrict the review to carbapenemase-producing organisms (CPE), excluding broader carbapenem-resistant (CRE) studies, requires justification. There is a potential risk that the exclusion could lead to the omission of relevant economic evaluations where phenotypic resistance is the primary cost driver, regardless of the underlying genotype. It would be prudent to expand the search and include CRE especially given a gen AI tool will be used to speed things up and there are not many cost-effectiveness studies in this literature unless there is a compelling reason not to do this.	Thank you for this comment, we agree with your point. We will make our review more comprehensive and include CRGNB, regardless of their mechanism of resistance. Please refer to our "Background"; and "Methods and Analysis" sections.
You plan to to apply discount rates to aggregate monetary values whilst assuming health effects remain stable is inconsistent with standard health economic guidelines for time horizons exceeding one year. This plan needs revision to ensure that both costs and outcomes are discounted appropriately and consistently. Also the current plan to apply a discount rate to a reported aggregate cost is confusing, what are you trying to achieve given discounting is a function of time within the model's horizon and applying a flat discount rate to a static cost figure sounds mathematically meaningless?	Thank you for this comment. The goal of discounting is to estimate all results in the same currency year (2025 CAD) to be able to compare the incremental cost-effectiveness ratio. We modified the text in the "Data analysis and aggregation of results" section.
The proposed approach of averaging scores across three different assessment tools should be discouraged, as the proposed summary scores can mask critical methodological flaws in specific domains. Furthermore, applying the Cochrane Risk of Bias tool—designed for clinical trials—to decision-analytic models risks penalising high-quality modelling studies. I'd like to see how these concerns can be addressed	We will exclude all modelling and simulation studies. Please refer to "Comparators and designs (C)" and "Table 1" for changes.
The plan to synthesise findings from multiple countries using a single Dominance Ranking Matrix requires clarification regarding how differences in unit costs, currency purchasing power, and health system thresholds will be handled. Without adjusting for these structural variations,	For this review, DRM will be used only to judge if an intervention costs less, more or equally to the comparator within the study, while yielding effective outcomes.

direct ranking of ICERs across different jurisdictions are very likely to yield misleading conclusions which would diminish the utility of your review greatly.	

VERSION 2 - REVIEW

Reviewer 2

Name Manoukian, Sarkis

Affiliation Glasgow Caledonian University, School of Health and Life Sciences

Date 26-Jan-2026

COI

I have now reviewed the protocol for the second time. I note that the authors have broadened the scope of their review to CRGNB as a positive development. However, the authors have now massively reduced the utility of this review by removing modelling and simulation studies from the search. I do not understand why this has been done given that it was originally a strength of this study. There are many important modelling studies in this field that are going to be excluded if they have that restriction in the search algorithm. I'm not sure how many pure CEAs or CUAs they will find if they exclude computer simulations or other models. My suggestion is to go for flexibility because there are not many good examples of economic analysis in this literature. Perhaps you can include modelling studies but analyse them separately? It should depend a little bit on what you find but it's important you examine them too in my opinion.

General comments:

I do not see any limitations at the strength and limitations section on page 2.

On pages 9-10 Legend of figure it appears that modelling studies are still included? I suggest they are added back and this section's wording re-examined.

VERSION 2 - AUTHOR RESPONSE

Reviewer: 2

Dr. Sarkis Manoukian, Glasgow Caledonian University

Comments to the Author:

I have now reviewed the protocol for the second time. I note that the authors have broadened the scope of their review to CRGNB as a positive development. However, the authors have now massively reduced the utility of this review by removing modelling and simulation studies from the search. I do not understand why this has been done given that it was originally a strength of this study. There are many important modelling studies in this field that are going to be excluded if they have that restriction in the search algorithm. I'm not sure how many pure CEAs or CUAs they will find if they exclude computer simulations or other models. My suggestion is to go for flexibility because there are not many good examples of economic analysis in this literature. Perhaps you can include modelling studies but analyse them separately? It should depend a little bit on what you find but it's important you examine them too in my opinion.

We agree and have re-inserted modelling studies in the search.

General comments:

I do not see any limitations at the strength and limitations section on page 2.

Good point. We restricted the search to studies published in English or French, which means relevant evidence in other languages may have been missed. As with all systematic reviews, there is also the possibility of time-lag bias, as recent or ongoing studies may not yet be published or caught by the time we stop our search. Finally, variation in study design, outcome definitions, and reporting across included studies may limit comparability and quantitative synthesis. We have added these limitations to the paper.

On pages 9-10 Legend of figure it appears that modelling studies are still included? I suggest they are added back and this section's wording re-examined.

We overlooked this, however we agree that they be added back. The legend now reflects the fact that we are including them.

VERSION 3 - REVIEW

**Reviewer**	**2**
**Name**	**Manoukian, Sarkis**
**Affiliation**	**Glasgow Caledonian University, School of Health and Life Sciences**
**Date**	**06-Feb-2026**

COI

Happy for this to be published in its current format
